# Heart Rate Variability Time-Domain Analysis Across Glaucoma Subtypes

**DOI:** 10.3390/biomedicines13040893

**Published:** 2025-04-07

**Authors:** Yuto Yoshida, Hinako Takei, Misaki Ukisu, Keigo Takagi, Masaki Tanito

**Affiliations:** Department of Ophthalmology, Shimane University Faculty of Medicine, Izumo 693-8501, Japan; y-yoshida@juntendo.ac.jp (Y.Y.); kei918.oor@icloud.com (K.T.)

**Keywords:** time-domain heart rate variability, autonomic nervous system, primary open-angle glaucoma, exfoliation glaucoma, intraocular pressure

## Abstract

**Background/Objectives**: The association between glaucoma and autonomic nervous system (ANS) function remains unclear. Primary open-angle glaucoma (POAG) and exfoliation glaucoma (EXG) have distinct pathophysiological mechanisms, which may lead to different ANS alteration. This study aimed to investigate the association between different glaucoma subtypes and the following time-domain heart rate variability (HRV) parameters: the standard deviation of normal-to-normal intervals (SDNN), the square root of the mean of the sum of the squared differences between adjacent normal-to-normal intervals (RMSSD), and the coefficient of variation of R-R intervals (CVRR). **Methods**: A total of 809 eyes from 809 patients with POAG, EXG, and controls were included. HRV was measured from the participants’ fingertips using a sphygmograph (TAS9 Pulse Analyzer Plus View; YKC Corp., Tokyo, Japan). Comparisons of time-domain HRV parameters among the groups were conducted. To evaluate the associations between time-domain HRV parameters and other variables, linear regression analyses were conducted. **Results**: This study included 522 participants with POAG, 191 participants with EXG, and 96 participants in the control group. There was a significant difference in CVRR among the groups (the control group: 4.04 ± 2.08%, the POAG group: 3.86 ± 1.87%, the EXG group: 3.57 ± 2.02%; *p* = 0.010), whereas no significant differences were found in SDNN and RMSSD. The EXG group had significantly lower values of SDNN and CVRR values compared to the POAG group (*p* = 0.0156 and *p* = 0.0037, respectively). In multivariate linear regression analysis, the highest recorded intraocular pressure (IOP) was significantly associated with CVRR. **Conclusions**: HRV parameters may reflect ANS alterations in glaucoma subtypes.

## 1. Introduction

Heart rate variability (HRV) is a non-invasive indicator that reflects the balance between sympathetic and parasympathetic activity within the autonomic nervous system (ANS) [1,2,3,4,5]. Time-domain HRV refers to the fluctuation in the intervals between consecutive heartbeats, quantified by measuring the R-R intervals of the QRS complex on an electrocardiogram [6]. As a reliable marker of ANS function, HRV has been widely used in cardiovascular research [7,8]. Additionally, HRV has been associated with various conditions, including neurodegenerative diseases such as Parkinson’s disease [9] and dementia [10], metabolic diseases [11,12], and psychiatric disorders [13]. Thus, HRV may be a potential indicator not only for cardiovascular disease but also for various other health conditions.

Glaucoma may be associated with alterations in HRV. Several studies have reported a relationship between ANS dysfunction and the presence of glaucoma [14,15,16,17]. Liu et al. demonstrated that lower HRV was associated with more rapid progression of glaucoma [16]. Intraocular pressure (IOP), a key factor influencing glaucoma progression, may be regulated by the ANS through its effects on the balance of aqueous humor production and outflow [18]. Additionally, glaucoma progression is associated with vascular risk factors, including blood pressure [19] and ocular perfusion pressure [20,21], which may also be modulated by the ANS [18]. Although these findings suggest a potential association between glaucoma and ANS function, the available evidence remains limited. Therefore, further research is needed to elucidate the underlying mechanisms.

Most previous studies investigating the association between glaucoma and HRV had limited sample sizes [16,22,23]. While some studies have focused on primary open-angle glaucoma (POAG) [14,15,16] or normal-tension glaucoma (NTG) [22,23], there are no studies investigating the association between HRV and exfoliation glaucoma (EXG). Given the high prevalence of EXG among the elderly and its substantial risk of blindness and visual impairment, examining its relationship with ANS dysfunction is crucial. Given these knowledge gaps, this study aims to investigate the association between HRV and different types of glaucoma, including POAG and EXG, using a larger sample size.

## 2. Materials and Methods

### 2.1. Participants and Study Design

This study was conducted according to the recommendations of the Declaration of Helsinki and was approved by the institutional review board of Shimane University Hospital (No.20200228-2, revised version issued on 27 October 2024). This cross-sectional study investigates the association between different types of glaucoma and the ANS parameters assessed via time-domain HRV. Participants were recruited from Shimane University between June 2023 and July 2024 and included patients with POAG and EXG, and individuals without any ocular diseases other than cataracts (control group). Instead of obtaining signed consent from participants, information about the study was published on the participating institution’s website, and an opt-out opportunity was provided. Exclusion criteria included (1) an HRV reliability score below 95% and (2) the presence of ocular diseases other than POAG, EXG, and cataract. In this study, one eye per participant was selected for analysis. For the control group, the eye with better visual acuity was chosen, and in cases where visual acuity was the same, the right eye was selected. For participants with POAG or EXG, the eye with the condition in unilateral cases was selected. For cases with bilateral glaucoma, the eye with the higher recorded IOP was selected; if both eyes had the same IOP, the right eye was chosen. In this study, glaucoma diagnosis was confirmed through clinical examination by ophthalmologists based on comprehensive ocular assessments, including IOP measurement, gonioscopy, optic nerve assessment by using fundus camera and optical coherence tomography, and visual field testing.

### 2.2. Time-Domain Heart Rate Variability

HRV was measured from the participants’ fingertips using a sphygmograph (TAS9 Pulse Analyzer Plus View; YKC Corp., Tokyo, Japan) in the HRV measurement mode. HRV measurements were recorded over a 20 s period at a sampling rate of 1 kHz while participants remained seated at rest. Additionally, all data were collected during daytime outpatient visits. In this study, the following parameters were measured with time-domain analysis: heart rate (HR), the standard deviation of normal-to-normal intervals (SDNN), the square root of the mean of the sum of the squared differences between adjacent normal-to-normal intervals (RMSSD), and the coefficient of variation of R-R intervals (CVRR). These parameters are utilized as indicators of parasympathetic nervous system activity. All measurements were conducted by experienced orthoptists trained in the procedure.

### 2.3. Other Covariates

In this study, the following demographic data were collected: age, sex, body mass index (BMI), systolic blood pressure (sBP), diastolic blood pressure (dBP), presence of hypertension and diabetes mellitus, and smoking history. The following ophthalmic parameters were collected from medical records: best-corrected visual acuity (BCVA), spherical equivalent refraction (SERE), mean deviation (MD) of the visual field (central 30-2 program, Humphrey Visual Field Analyzer, Carl Zeiss Meditec, Dublin, CA, USA), current IOP, highest recorded IOP, and the presence of pseudophakia. Decimal BCVA values were converted to the logarithm of the minimum angle of resolution (logMAR), with counting fingers, hand motions, light perception, and no light perception assigned decimal values of 0.0025, 0.002, 0.0016, and 0.0013, respectively [24].

### 2.4. Statistical Analysis

Continuous variables were presented as mean ± standard deviation (SD), while categorical variables were presented as percentages. Categorical variables were compared among the three groups using the Chi-square test. Continuous variables were analyzed using the Kruskal–Wallis test. Comparisons of time-domain HRV parameters among the POAG, EXG, and control groups were also performed using the Kruskal–Wallis test to assess intergroup differences. For post hoc analyses, pairwise comparisons were conducted using the Mann–Whitney U test with Bonferroni correction to adjust for multiple comparisons. To evaluate the associations between time-domain HRV parameters and other variables, both simple and multivariate linear regression analyses were conducted. The covariates known to influence HRV were included in the regression models [3,7,8,11,12,16,18]. Model 1 was adjusted for age, sex, glaucoma type, sBP, dBP, HR, current IOP, BMI, diabetes mellitus, and smoking history, whereas Model 2 was adjusted for age, sex, glaucoma type, sBP, dBP, HR, highest recorded IOP, BMI, diabetes mellitus, and smoking history. False discovery rate (FDR) correction using the Benjamini–Hochberg procedure was also applied to control for the potential inflation of type I errors. All statistical analyses were carried out using STATA/SE 15.0 for Mac (StataCorp, College Station, TX, USA), with statistical significance set at *p* < 0.05.

## 3. Results

**Figure 1:**
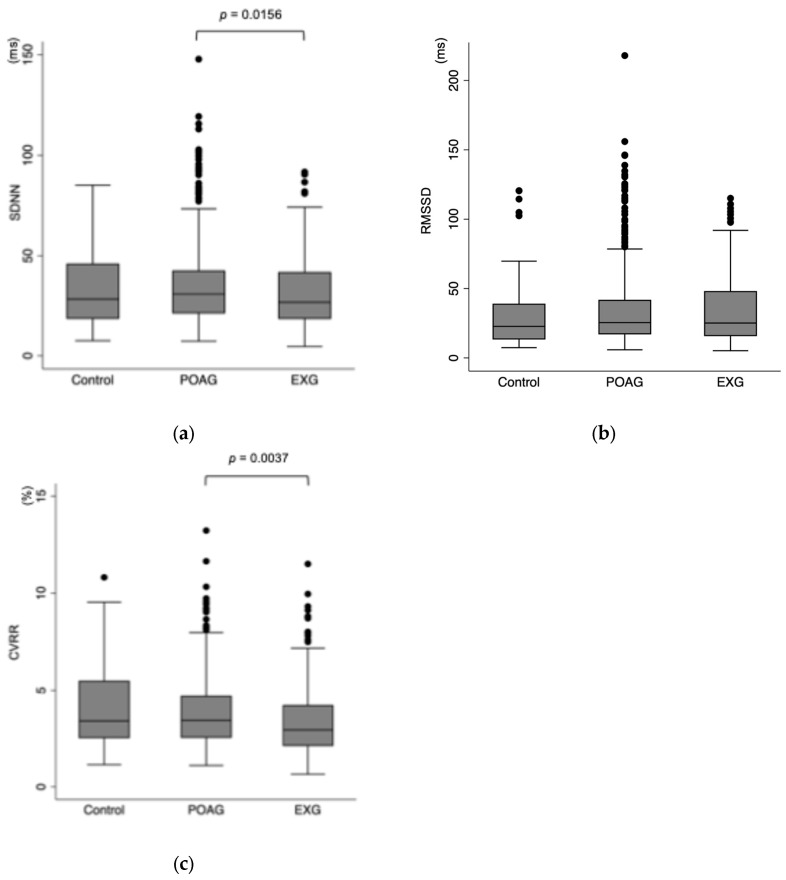
Association between time-domain parameters and glaucoma. (**a**) SDNN in control, POAG, and EXG were 33.6 ± 18.7 ms, 35.3 ± 20.1 ms, and 32.0 ± 18.5 ms, respectively (Kruskal–Wallis test, *p* = 0.051). In terms of SDNN, a Mann–Whitney test with Bonferroni correction showed a significant difference between POAG and EXG (*p* = 0.0156). (**b**) RMSSD in control, POAG, and EXG were 30.6 ± 25.2 ms, 35.1 ± 28.5 ms, and 34.7 ± 24.5 ms, respectively (Kruskal–Wallis test, *p* = 0.132). (**c**) CVAA in control, POAG, and EXG were 4.04 ± 2.08%, 3.86 ± 1.87%, and 3.57 ± 2.02%, respectively (Kruskal–Wallis test, *p* = 0.010). In terms of CVRR, a Mann–Whitney test with Bonferroni correction showed a significant difference between POAG and EXG (*p* = 0.0037). Post hoc comparisons were also performed using the Mann–Whitney U test with Bonferroni correction (SDNN: Control vs. POAG, *p* = 0.3656; Control vs. EXG, *p* = 0.4918; RMSSD: Control vs. POAG, *p* = 0.0453; Control vs. EXG, *p* = 0.1056; CVRR: Control vs. POAG, *p* = 0.6851; Control vs. EXG, *p* = 0.1056). Bonferroni-corrected significance threshold was set at *p* < 0.0167. POAG = primary open-angle glaucoma; EXG = exfoliation glaucoma; SDNN = the standard deviation of normal-to-normal intervals; RMSSD = the square root of the mean of the sum of the squared differences between adjacent normal-to-normal intervals; CVRR = the coefficient of variation of R-R intervals.

A total of 809 eyes (809 participants) were included in this study, comprising 522 participants with POAG, 191 participants with EXG, and 96 participants in the control group. Table 1 summarizes the participant characteristics. The mean age ± SD was 66.0 ± 12.7 years in the POAG group, 75.8 ± 8.7 years in the EXG group, and 59.9 ± 18.8 years in the control group. Figure 1 shows the association between time-domain HRV parameters and different types of glaucoma. SDNN values in the control, POAG, and EXG groups were 33.6 ± 18.7 ms, 35.3 ± 20.1 ms, and 32.0 ± 18.5 ms, respectively (Kruskal–Wallis test, *p* = 0.051), suggesting a borderline association. Post hoc pairwise comparisons using the Mann–Whitney U test revealed a significant difference between the POAG and EXG groups (*p* = 0.0156). RMSSD values in the control, POAG, and EXG groups were 30.6 ± 25.2 ms, 35.1 ± 28.5 ms, and 34.7 ± 24.5 ms, respectively, with no significant differences among the groups (*p* = 0.132). CVRR values in the control, POAG, and EXG groups were 4.04 ± 2.08%, 3.86 ± 1.87%, and 3.57 ± 2.02%, respectively (*p* = 0.010). Post hoc analysis identified a significant difference between the POAG and EXG groups (*p* = 0.0037).

Table 2 presents the multiple regression models examining the associations between SDNN and various factors. In the multiple linear regression models (Model 1 and Model 2), significant associations were observed with age (≥75 years), HR, and BMI. In contrast, there were no significant associations with POAG or EXG.

Table 3 shows the associations between RMSSD and various factors. In both Model 1 and Model 2, RMSSD was significantly associated with HR and BMI, whereas no significant associations were observed with age or glaucoma.

Table 4 presents the associations between CVRR and various factors. CVRR was significantly lower in older adults (65–74 years and ≥75 years) compared to those younger than 65 years. In Model 1 and 2, CVRR showed significant associations with HR and BMI. In the unadjusted analysis, EXG was significantly associated with lower CVRR (coef, –0.48; 95% confidence interval (CI), −0.95 to –0.003; *p* = 0.048); however, in Model 2, this association was at the borderline level (coef, −0.50; 95%CI: −1.03 to −0.03; *p* = 0.065). In multivariate linear regressions, the highest recorded IOP was significantly associated with CVRR (coef, 0.01, 95%CI: 0.001 to 0.03; *p* = 0.050), whereas there was no significant association between the current IOP and CVRR.

After applying FDR correction, significant associations were observed between HR and all HRV parameters including SDNN, RMSSD, and CVRR. Additionally, a significant association was identified between CVRR and Age ≥ 75.

## 4. Discussion

This study is a cross-sectional study investigating the association between HRV and different types of glaucoma, including POAG and EXG. The time-domain HRV parameters, such as SDNN and CVRR, showed significant differences between the POAG and EXG groups, while RMSSD showed no significant differences among the groups. In multivariate linear regression analyses, CVRR showed a borderline association with EXG, while the highest recorded IOP was significantly associated with CVRR.

To the best of our knowledge, this study is the first to demonstrate the associations between various time-domain HRV parameters and glaucoma or IOP with a large sample size. A previous study by Asefa et al. was a population-based cohort study investigating the association between HRV and glaucoma in 86,841 participants [25]; however, the definition of glaucoma was based on self-reported glaucoma diagnosis and treatment in combination with the NEI-VFQ-25 questionnaire. As glaucoma diagnosis was confirmed through clinical examination in this study, our findings may provide more robust evidence regarding the association between HRV and glaucoma. Additionally, previous studies did not comprehensively examine time-domain HRV parameters: Liu et al. focused on SDNN [16], Asefa et al. on RMSSD [25], and Kurysheva et al. on both SDNN and RMSSD [22,23]. In contrast, our study examined various parameters including SDNN, RMSSD, and CVRR, thereby providing a more comprehensive investigation of the association between glaucoma and time-domain HRV parameters.

Patients with EXG may demonstrate more significant ANS dysregulation compared to those with POAG or individuals without glaucoma. In this study, HRV parameters, such as SDNN and CVRR, were lower in the EXG group compared to the POAG and control groups (SDNN: 33.6 ± 18.7 ms, 35.3 ± 20.1 ms, and 32.0 ± 18.5 ms; CVRR: 4.04 ± 2.08%, 3.86 ± 1.87%, and 3.57 ± 2.02% in the control, POAG, and EXG groups, respectively). Exfoliation syndrome (XFS), which is the cause of EXG, has been linked to vascular dysfunction through the following mechanisms: impaired vascular endothelial function [26], increased ocular and systemic antioxidant stress [27,28], and abnormality of clot formation [29], all of which may contribute to the ANS dysfunction. Additionally, Visontai et al. reported that various HRV parameters, including SDNN and RMSSD, were lower in patients with XFS than in the control group [30]. However, in this study, the differences among groups became less pronounced after adjusting for age. This finding may reflect the higher prevalence of EXG in older individuals. Therefore, given that EXG is more common in older individuals, the frequency of ANS dysfunction may be higher in patients with EXG. Further research is needed to examine the association between EXG and the ANS function.

Moreover, time-domain HRV parameters may be associated with IOP. According to a previous study, IOP may be regulated by the ANS through the production and outflow of aqueous humor [18]. In our study, no significant association between the current IOP and HRV was observed, whereas the highest recorded IOP was significantly associated with CVRR. Liu et al. reported that there was a significant difference in the IOP fluctuation between the lowest HRV group and the highest HRV group [16]. Therefore, future studies should investigate not only IOP but also IOP fluctuations to explore their association with HRV. Moreover, our study found a positive correlation between the highest recorded IOP and CVRR (coef, 0.01, 95%CI: 0.001 to 0.03; *p* = 0.050). The ANS may play a crucial role in the short-term regulation of IOP, similar to BP [31,32,33]. Therefore, transiently elevated IOP may lead to an increase in parasympathetic nervous activity. Future research should investigate the relationship between IOP and the ANS balances.

In this study, time-domain HRV parameters were significantly associated with age, HR, and BMI. These findings were consistent with previous research [34,35,36,37,38,39]. Especially, numerous studies have reported that HRV decreases with advancing age [38,39,40,41,42], suggesting that HRV may be related to aging processes. Therefore, further research is warranted to explore whether HRV plays a role in the development of age-related ocular diseases, including glaucoma.

This study has several limitations. First, the sample size of the control group was smaller compared to the POAG and EXG groups, which may have influenced the results. Second, there may be a selection bias. Since this study included patients who visited the university hospital, the control group may not represent the healthy population. Third, there is a potential for measurement bias in HRV. The sphygmograph used in this study did not allow access to raw RR interval data, and artifact editing could not be performed. The limited recording duration of 20 s in this study may reduce the generalizability of the findings. Nevertheless, HRV is a well-established measurement with moderate-to-good reliability [43], suggesting that the results of this study remain meaningful. Fourth, the use of antiglaucoma medications or antihypertensive drugs was not considered in this study. These medications may have influenced IOP, sBP, dBP, and HRV. Fifth, this study is a cross-sectional analysis; therefore, it does not establish a causal relationship between glaucoma subtypes and the autonomic nervous system. To determine whether a causal link exists, future longitudinal studies will be necessary. Sixth, due to its retrospective design, we were unable to control or obtain detailed information on potential confounding factors that may influence ANS activity, such as recent caffeine intake, physical activity, emotional state, or medication use.

## 5. Conclusions

This is the first study to show the relationship between various time-domain HRV parameters and glaucoma with a large sample size. The patients with EXG had significantly lower SDNN and CVRR values compared to those with POAG and the control group. Additionally, the highest recorded IOP was significantly associated with CVRR. In conclusion, HRV parameters may reflect ANS alterations in glaucoma subtypes.

## Figures and Tables

**Table 1:** Participant characteristics.

Parameters	Control (n = 96)	POAG (n = 522)	EXG (n = 191)	*p* Value
Age, years	59.9 ± 18.8	66.0 ± 12.7	75.8 ± 8.7	<0.001
Age group, %				
<65	40 (41.7%)	204 (39.1%)	19 (9.9%)	<0.001
65−74	40 (41.7%)	188 (36.0%)	67 (35.1%)	
≥75	16 16.7%)	130 (24.9%)	105 (55.0%)	
Female, %	41 (42.7%)	237 (45.4%)	87 (45.5%)	0.880
Left eye, %	30 (31.3%)	182 (34.9%)	86 (45.0%)	0.022
Body mass index, kg/m², SD	23.2 ± 4.4	22.7 ± 3.3	22.8 ± 3.2	0.857
Systolic blood pressure, mmHg, SD	141.2 ± 23.4	140.8 ± 20.8	148.2 ± 20.2	<0.001
Diastolic blood pressure, mmHg, SD	79.5 ± 14.3	80.8 ± 13.1	80.3 ± 14.1	0.591
Heart rate, beats/minute, SD	75.1 ± 12.5	68.2 ± 11.4	68.8 ± 12.3	<0.001
BCVA, LogMAR, SD	−0.01 ± 0.1	0.15 ± 0.4	0.44 ± 0.8	<0.001
SERE, D, SD	−1.85 ± 3.3	−3.41 ± 3.7	−1.70 ± 2.8	<0.001
MD, dB, SD	−1.37 ± 2.8	−6.37 ± 7.2	−7.05 ± 7.4	<0.001
IOP (current), mmHg, SD	15.1 ± 3.6	15.4 ± 6.2	19.3 ± 9.9	<0.001
IOP (highest recorded), mmHg, SD	16.5 ± 3.5	22.4 ± 9.7	28.1 ± 10.5	<0.001
Pseudophakia, %	28 (29.1%)	257 (49.2%)	147 (77.0%)	<0.001
Hypertension, %	28 (29.1%)	225 (43.1%)	96 (50.3%)	0.003
Diabetes, %	20 (20.8%)	69 (13.2%)	31 (16.2%)	0.128
Smoking, %	12 (16.3%)	56 (11.3%)	21 (11.3%)	0.375

SD = standard deviation; D = diopters; dB = decibels; IOP = intraocular pressure; logMAR = logarithm of the minimum angle of resolution; MD = mean deviation; SERE = sphere equivalent refractive error; BCVA = best corrected visual acuity.

**Table 2:** Associations between SDNN and various factors.

	Unadjusted Model		Model 1		Model 2	
	Coef (95% CI)	*p* Value	Coef (95% CI)	*p* Value	Coef (95% CI)	*p* Value
Age						
<65	Ref	−	Ref	−	Ref	−
65−74	−1.83 (−5.09, 1.43)	0.271	−2.03 (−5.44, 1.38)	0.243	−1.96 (−5.37, 1.45)	0.259
≥ 75	−3.35 (−6.74, 0.04)	0.053	−4.11 (−8.03, −0.19)	0.040	−3.98 (−7.90, −0.05)	0.047
Sex						
Male	Ref	−	Ref	−	Ref	−
Female	−0.28 (−3.00, 2.44)	0.842	0.78 (−2.02, 3.59)	0.583	0.96 (−1.85, 3.78)	0.502
Glaucoma type						
Control	Ref	−	Ref	−	Ref	−
POAG	1.76 (−2.50, 6.03)	0.417	−1.03 (−5.28, 3.23)	0.636	−1.80 (−6.15, 2.54)	0.415
EXG	−1.55 (−6.35, 3.26)	0.528	−2.66 (−7.61, 2.29)	0.292	−3.62 (−8.79, 1.55)	0.170
sBP	−0.08 (−0.15, −0.02)	0.013	0.001 (−0.09, 0.10)	0.988	−0.002 (−0.10, 0.09)	0.964
dBP	−0.09 (−0.19, 0.02)	0.103	0.01 (−0.14, 0.15)	0.940	0.01 (−0.13, 0.16)	0.862
Heart rate	−0.51 (−0.61, −0.41)	**0.000**	−0.52 (−0.62, −0.41)	**0.000**	−0.52 (−0.63, −0.42)	**0.000**
IOP (current)	0.08 (−0.11, 0.26)	0.423	0.15 (−0.04, 0.34)	0.119	−	−
IOP (highest recorded)	0.08 (−0.06, 0.21)	0.251	−	−	0.14 (−0.01, 0.28)	0.060
Body mass index	−0.57 (−0.98, −0.17)	**0.006**	−0.45 (−0.85, −0.05)	0.029	−0.41 (−0.81, −0.01)	0.045
Diabetes	−3.16 (−6.96, 0.64)	0.103	−0.44 (−4.27, 3.39)	0.822	−0.49 (−4.32, 3.34)	0.801
Smoking	0.10 (−4.18, 4.38)	0.963	0.42 (−3.84, 4.68)	0.847	0.32 (−3.93, 4.57)	0.882

*p*-values that remained significant following false discovery rate (FDR) correction are highlighted in bold. SDNN = the standard deviation of normal-to-normal intervals; POAG = primary open-angle glaucoma; EXG = exfoliation glaucoma; IOP = intraocular pressure; BP = blood pressure; Coef = coefficient; CI = confidence interval; Ref = reference.

**Table 3:** Associations between RMSSD and various factors.

	Unadjusted Model		Model 1		Model 2	
	Coef (95% CI)	*p* Value	Coef (95% CI)	*p* Value	Coef (95% CI)	*p* Value
Age						
<65	Ref	−	Ref	−	Ref	−
65−74	4.09 (−0.46, 8.65)	0.078	3.51 (−1.27, 8.30)	0.149	3.62 (−1.16, 8.40)	0.137
≥75	6.32 (1.58, 11.1)	0.009	4.76 (−0.73, 10.3)	0.089	5.00 (−0.51, 10.5)	0.075
Sex						
Male	Ref	−	Ref	−	Ref	−
Female	−1.78 (−5.58, 2.03)	0.360	0.36 (−3.57, 4.30)	0.855	0.61 (−3.33, 4.56)	0.761
Glaucoma type						
Control	Ref	−	Ref	−	Ref	−
POAG	4.49 (−1.50, 10.5)	0.141	0.17 (−5.79, 6.13)	0.955	−0.87 (−6.95, 5.21)	0.779
EXG	4.03 (−2.71, 10.8)	0.241	−0.83 (−7.77, 6.11)	0.814	−2.37 (−9.61, 4.86)	0.520
Systolic BP	−0.07 (−0.16, 0.03)	0.156	−0.01 (−0.14, 0.13)	0.937	−0.01 (−0.14, 0.12)	0.892
Diastolic BP	−0.15 (−0.30, −0.01)	0.042	−0.01 (−0.21, 0.20)	0.953	0.001 (−0.20, 0.21)	0.989
Heart rate	−0.72 (−0.86, −0.58)	**0.000**	−0.70 (−0.85, −0.56)	**0.000**	−0.71 (−0.86, −0.56)	**0.000**
IOP (current)	0.09 (−0.18, 0.35)	0.515	0.13 (−0.13, 0.40)	0.320	−	−
IOP (highest recorded)	0.14 (−0.04, 0.33)	0.135	−	−	0.18 (−0.02, 0.38)	0.080
Body mass index	−0.83 (−1.40, −0.26)	**0.004**	−0.62 (−1.19, −0.06)	0.031	−0.58 (−1.15, −0.02)	0.042
Diabetes	0.40 (−4.94, 5.73)	0.885	2.57 (−2.80, 7.93)	0.348	2.47 (−2.89, 7.83)	0.366
Smoking	2.86 (−3.12, 8.84)	0.349	5.38 (−0.59, 11.3)	0.077	5.30 (−0.65, 11.3)	0.081

*p*-values that remained significant following false discovery rate (FDR) correction are highlighted in bold. RMSSD = the square root of the mean of the sum of the squared differences between adjacent normal-to-normal intervals; POAG = primary open-angle glaucoma; EXG = exfoliation glaucoma; IOP = intraocular pressure; BP = blood pressure; Coef = coefficient; CI = confidence interval; Ref = reference.

**Table 4:** Associations between CVRR and various factors.

	Unadjusted Model		Model 1		Model 2	
	Coef (95% CI)	*p* Value	Coef (95% CI)	*p* Value	Coef (95% CI)	*p* Value
Age						
<65	Ref	−	Ref	−	Ref	−
65−74	−0.48 (−0.80, −0.16)	**0.003**	−0.41 (−0.76, −0.06)	0.023	−0.40 (−0.75, −0.05)	0.025
≥75	−0.64 (−0.97, −0.30)	**0.000**	−0.63 (−1.04, −0.23)	**0.002**	−0.62 (−1.02, −0.22)	**0.003**
Sex						
Male	Ref	−	Ref	−	Ref	−
Female	0.09 (−0.18, 0.36)	0.505	0.09 (−0.19, 0.38)	0.518	0.11 (−0.18, 0.40)	0.439
Glaucoma type						
Control	Ref	−	Ref	−	Ref	−
POAG	−0.19 (−0.61, 0.23)	0.383	−0.29 (−0.72, 0.15)	0.197	−0.37 (−0.82, 0.08)	0.103
EXG	−0.48 (−0.95, −0.003)	0.048	−0.40 (−0.91, 0.11)	0.125	−0.50 (−1.03, −0.03)	0.065
Systolic BP	−0.01 (−0.02, −0.002)	**0.007**	−0.002 (−0.01, 0.01)	0.689	−0.002 (−0.01, 0.01)	0.643
Diastolic BP	−0.01 (−0.02, 0.003)	0.176	0.0002 (−0.02, 0.01)	0.981	0.0001 (−0.01, 0.02)	0.936
Heart rate	−0.02 (−0.03, −0.01)	**0.000**	−0.02 (−0.03, −0.01)	**0.000**	−0.02 (−0.03, −0.01)	**0.000**
IOP (current)	0.01 (−0.01, 0.03)	0.223	0.02 (−0.003, 0.04)	0.095	−	−
IOP (highest recorded)	0.01 (−0.01, 0.02)	0.222	−	−	0.01 (0.001, 0.03)	0.050
Body mass index	−0.05 (−0.09, −0.01)	0.011	−0.05 (−0.09, −0.01)	0.024	−0.04 (−0.08, −0.002)	0.038
Diabetes	−0.28 (−0.66, 0.09)	0.138	−0.11 (−0.49, 0.30)	0.646	−0.10 (−0.49, 0.30)	0.627
Smoking	−0.06 (−0.48, 0.36)	0.772	−0.11 (−0.54, 0.33)	0.631	−0.12 (−0.55, 0.32)	0.597

*p*-values that remained significant following false discovery rate (FDR) correction are highlighted in bold. CVRR = the coefficient of variation of R-R intervals; POAG = primary open-angle glaucoma; EXG = exfoliation glaucoma; IOP = intraocular pressure; BP = blood pressure; Coef = coefficient; CI = confidence interval; Ref = reference.

## Data Availability

The raw data supporting the conclusions of this article will be made available by the authors on request.

## Abbreviations

The following abbreviations are used in this manuscript:

HRV	Heart rate variability
ANS	Autonomic nervous system
SDNN	The standard deviation of normal-to-normal intervals
RMSSD	The square root of the mean of the sum of the squared differences between adjacent normal-to-normal intervals
CVRR	the coefficient of variation of R-R intervals
POAG	Primary open-angle glaucoma
EXG	Exfoliation glaucoma
NTG	Normal-tension glaucoma
IOP	Intraocular pressure
BMI	Body mass index
BP	Blood pressure
BCVA	Best-corrected visual acuity
SERE	Spherical equivalent refraction
MD	Mean deviation
LogMAR	Logarithm of the minimum angle of resolution
CI	Confidence interval
FDR	False discovery rate
